# The Influence of Calcification Factors and Endothelial-Dysfunction Factors on the Development of Unstable Atherosclerotic Plaques

**DOI:** 10.3390/diagnostics10121074

**Published:** 2020-12-11

**Authors:** Yana V. Polonskaya, Elena V. Kashtanova, Ivan S. Murashov, Aleksei V. Kurguzov, Evgeny V. Sadovski, Nikolay A. Maslatsov, Ekaterina M. Stakhneva, Alexander M. Chernyavskii, Yuliya I. Ragino

**Affiliations:** 1 Research Institute of Internal and Preventive Medicine, Branch of the Institute of Cytology and Genetics, Siberian Branch of Russian Academy of Sciences, Bogatkova Str. 175/1, 630089 Novosibirsk, Russia; yana-polonskaya@yandex.ru (Y.V.P.); elekastanova@yandex.ru (E.V.K.); stinger000@mail.ru (E.V.S.); maslatsoff@mail.ru (N.A.M.); ragino@mail.ru (Y.I.R.); 2 The Federal State Budgetary Institution “National Medical Research Center named Academician E.N. Meshalkin” of the Ministry of Health of the Russian Federation, 630055 Novosibirsk, Russia; ivmurashov@gmail.com (I.S.M.); aleksey_kurguzov@mail.ru (A.V.K.); amchern@mail.ru (A.M.C.)

**Keywords:** calcification of atherosclerotic plaques, unstable atherosclerotic plaque, osteonectin, osteopontin, osteocalcin, E-selectin, sVCAM

## Abstract

Background: This study aimed to evaluate changes in markers of calcification and of endothelial dysfunction during the development of calcification and instability of atherosclerotic plaques and to identify associations of calcification factors with the formation of unstable plaques. Methods: We analyzed 44 male patients with coronary atherosclerosis who underwent endarterectomy in coronary arteries during coronary bypass surgery. The endarterectomy material (intima/media) was examined using histological and biochemical methods, and the stability and calcification degree of atherosclerotic plaques were assessed. In homogenates of the tissue samples and in blood, concentrations of osteoprotegerin, osteocalcin, osteopontin, osteonectin, monocyte-chemoattractant protein type 1 (MCP-1), soluble vascular cell adhesion molecule 1 (sVCAM-1), and E-selectin were determined by enzyme immunoassays. Results: Unstable atherosclerotic plaques proved to be calcified more frequently (80.4% of plaques) than stable ones (45.0%). Osteonectin, E-selectin, and sVCAM-1 levels were lower in unstable plaques and plaques with large calcification deposits. Osteocalcin content increased with the increasing size of the calcification deposits in plaque. Blood osteocalcin concentration directly correlated with osteocalcin concentration in atherosclerotic plaques and was higher in the blood of patients with calcified plaques in coronary arteries. Conclusions: The results provide the basis for further research on the suitability of osteocalcin as a potential biomarker of an unstable calcified atherosclerotic plaque in a coronary artery.

## 1. Introduction

Coronary heart disease (CHD) is one of major causes of high morbidity and mortality worldwide [1]. The pathomorphological basis of CHD is coronary atherosclerosis, in which an important role is played by endothelial dysfunction and by the process of calcification, among others. The search for new biomarkers of atherosclerosis and of coronary artery calcification is actively being conducted.

Coronary calcification is linked with the risk of adverse cardiovascular events in patients with coronary atherosclerosis. For 15 years, Shaw et al. observed 9715 patients without clinical manifestations of CHD at baseline. The authors noted that, in patients with even a low degree of arterial calcification, the risk of mortality was almost 70% higher, whereas it was sixfold higher in patients with the largest calcification deposits in arteries than in patients who have no calcification deposits in arteries [2]. According to Criqui et al., the volume of coronary artery calcification is positively and independently associated with the risk of CHD, while calcification density at any volume of calcification is inversely proportional to the CHD risk [3]. Even though a higher overall coronary calcification index is a marker of increased cardiovascular risk, Puchner noticed that a low level of local calcium indicates instability of an atherosclerotic plaque, whereas a high calcium concentration with high density may be a marker of plaque stability [4].

Because of the abovementioned discrepancy, addressing the question of whether calcification indeed affects the formation of an unstable atherosclerotic plaque was chosen as one of the aims of this study. In addition, we investigated possible associations (including the joint effect) of calcification factors and of endothelial-dysfunction factors with the formation of calcified (including unstable) atherosclerotic plaques.

## 2. Materials and Methods

This study was approved by the local Ethics Committee of the Research Institute of Internal and Preventive Medicine—Branch of the Institute of Cytology and Genetics, Siberian Branch of Russian Academy of Sciences (protocol №2, approval on 3 July 2017). The study involved 108 male patients (mean age 60.6 ± 7.8 years) admitted to the Clinic of the Federal State Budgetary Institution “National Medical Research Center named academician E.N. Meshalkin” of the Ministry of Health of the Russian Federation for coronary bypass surgery. All the patients signed an informed consent form for participation in the study. The inclusion criteria were the absence of myocardial infarction less than 6 months old, the absence of acute chronic infectious and inflammatory diseases and their exacerbations, and the absence of renal failure, liver diseases, cancer, and hyperparathyroidism (Table 1). All patients received standard coronary artery disease therapy prior to coronary bypass surgery: statins, beta-blockers, angiotensin converting enzyme inhibitors, and disaggregants. In the 44 males who were chosen for further study, according to intraoperative indications during the coronary artery bypass operation, endarterectomy was performed in the coronary arteries.

**Figure 1:**
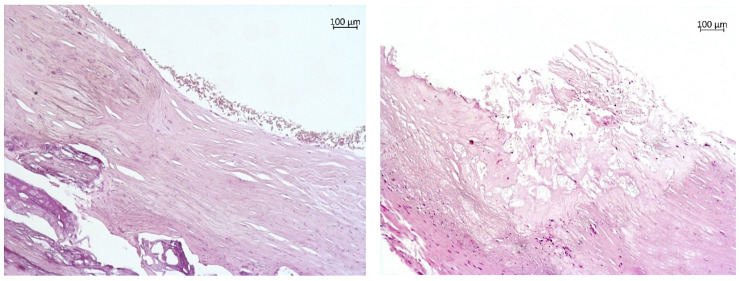
Atherosclerotic plaques of coronary arteries. (**a**) A stable fibrous plaque with calcification (magnification 100×; hematoxylin–eosin staining). A stable fibrous plaque is shown that has retained the integrity of the fibrous cap and of the endothelial lining. In the lumen, erythrocytes that have retained their integrity are visible. The large calcification deposits are present in the layers distal to the lumen. (**b**) An unstable atherosclerotic plaque (magnification 100×; hematoxylin–eosin staining). The plaque features a large atheromatous core composed of amorphous masses, cholesterol crystals, and extracellular lipids. A cap with well-pronounced disintegration and tearing is visible.

Each endarterectomy tissue sample was longitudinally and transversely divided symmetrically into several parts for histological and biochemical assays. Under an AxioLab.A1 binocular microscope (Carl Zeiss, Munich, Germany), an examination of 140 fragments of the coronary arteries was conducted, including the description of the cap, core, and periphery of each atherosclerotic plaque. In accordance with the results of the histological analysis, 89 samples of stable atherosclerotic plaques were identified (a thick plaque cap, a homogeneous lipid core <40% of plaque volume, and no inflammatory alterations, Figure 1a) and 51 samples of unstable plaques were identified (plaque cap thickness less than 65 μm, the lipid core >40% of the plaque volume, and >25 cell infiltration by macrophages and T lymphocytes in a field of view of a 0.3 mm diameter, Figure 1b).

**Figure 2:**
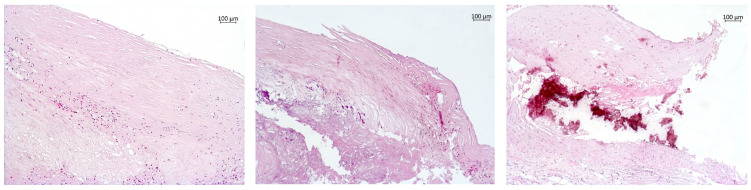
Atherosclerotic plaques with various degrees of calcification. (**a**) Without calcification deposits. A stable fibrous plaque with onset of atheromatosis is presented (magnification 100×; hematoxylin–eosin staining). The plaque has retained the integrity of the thick fibrous cap and endothelial lining. In the layers distal from the lumen, there are foci of foam cell infiltration and a small core composed of cholesterol crystals, extracellular lipids, and foam cells. (**b**) Small calcification is present in an unstable atherosclerotic plaque (magnification 100×; hematoxylin–eosin staining). An atherosclerotic plaque with a large atheromatous core consisting of amorphous masses, cholesterol crystals, and small-size calcification deposits. The fibrous cap is unevenly thinned and shows ruptures and areas of mononuclear infiltration. (**c**) Large calcification is present in an unstable atherosclerotic plaque (magnification 100×; hematoxylin–eosin staining). The plaque features areas of large-size calcification and a thick fibrous cap with ruptures in the shoulder region.

**Figure 3:**
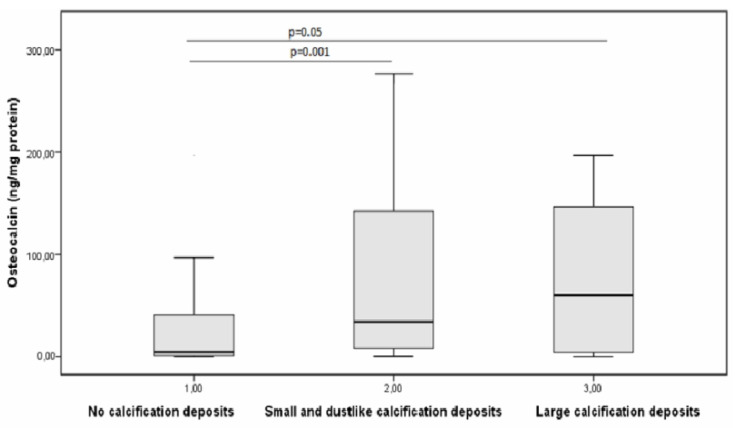
Changes in the concentration of osteocalcin with an increase in the degree of calcification of atherosclerotic plaque.

On the basis of the presence of calcification deposits, the plaque samples were subdivided into three groups: (1) 59 tissue samples without calcification deposits (Figure 2a), (2) 67 samples with small and dust-like calcification deposits (Figure 2b), and (3) 14 samples with large calcification deposits (Figure 2c).

Venous blood was collected from the patients before the surgical operation, 12 h after a meal. For biochemical assays, 1% homogenates in PBS (phosphate buffered saline) were prepared from intima/media samples of coronary arteries. In the intima/media homogenates, protein was quantified using the Lowry method. In the homogenates of intima/media and in the blood samples, concentrations of osteoprotegerin, osteopontin, monocyte-chemoattractant protein type 1 (MCP-1), soluble vascular cell adhesion molecule 1 (sVCAM-1), E-selectin (enzyme immunoassays from Bender MedSystems, Vienna, Austria), osteocalcin, and osteonectin (enzyme immunoassays from Immunodiagnostic Systems Ltd., Bensheim, Germany) were determined on a Multiscan EX (Thermo Electron Corporation, Vantaa, Finland). All results on the intima/media homogenates were normalized to the protein content of the samples.

The results were statistically analyzed using the SPSS software (version 17.0, USA). The normality of distribution of biomarker levels was determined using the Kolmogorov-Smirnov test. These distributions were not normal; therefore, nonparametric tests were applied. The results are presented as the 25th, 50th, and 75th percentiles. The significance of differences was evaluated using the Mann-Whitney test and chi-square test for categorical variables. Multiple comparisons among the groups were performed with the Kruskal-Wallis method. Univariate correlation (Spearman’s) analysis and multivariate linear regression and logistic regression analyses were carried out to find independent predictors of unstable plaques and calcified plaques. Data were considered statistically significant at *p* < 0.05.

## 3. Results

Analysis of histological data showed that 80.4% of unstable plaques were calcified (60.8% contained small and dust-like calcification deposits, and 19.6% contained large calcification deposits), as were 45.0% of stable plaques (40.5% contained small and dust-like calcification deposits, and 4.5% contained large calcification deposits).

Comparative analysis of calcification factors and endothelial-dysfunction factors between stable and unstable plaques revealed significant differences only in three markers (Table 2). In unstable plaques, the levels of osteonectin, sVCAM-1, and E-selectin were 1.5-, 1.8-, and 2.7-fold lower, respectively, in comparison with stable plaques.

A comparative analysis of the calcification factors and endothelial-dysfunction factors among different stages of plaque calcification (according to histological analysis) uncovered significant differences in five of the seven studied markers (Table 3). Levels of calcification factors osteopontin and osteonectin decreased (more than fourfold and twofold, respectively) with the increasing plaque calcification (plaques without calcification → plaques with small and dustlike calcification deposits → plaques with large calcification deposits) and were the lowest in plaques with large calcification deposits. Similarly, concentrations of endothelial-dysfunction factors—sVCAM-1 and E-selectin—decreased more than threefold and fivefold, respectively.

The opposite result was obtained for the calcification-related substance osteocalcin. Specifically, the level of osteocalcin increased with the progressing plaque calcification and was the highest in plaques with large calcification deposits (10.7-fold higher than its concentration in plaques without calcification) (Figure 3).

The analysis of relationships between calcification indicators and markers of endothelial dysfunction in the atherosclerotic foci revealed direct moderate correlations between osteopontin and sVCAM-1 (*r* = 0.388, *p* = 0.001) and MCP-1 (*r* = 0.523, *p* = 0.0001) and between osteonectin and sVCAM-1 (*r* = 0.669, *p* = 0.0001), MCP-1 (*r* = 0.421, *p* = 0.0001), and E-selectin (*r* = 0.520, *p* = 0.001). Multivariate linear regression analysis—where one of the calcification factors served as a dependent variable, and markers of endothelial dysfunction served as independent variables—also yielded significant results. It was found that osteonectin is associated with sVCAM-1 (B = 0.01, *p* = 0.0001) and E-selectin (B = 0.055, *p* = 0.001), *R*^2^ = 0.596, *p* = 0.0001, osteopontin is associated with sVCAM-1 (B = 0.013, *p* = 0.043), *R*^2^ = 0.296, *p* = 0.0001, and osteoprotegerin with sVCAM-1 (B = 0.249, *p* = 0.038), *R*^2^ = 0.084, *p* = 0.014. These results confirmed the unidirectionality of changes in the levels of osteopontin and osteonectin with respect to the changes in the levels of endothelial-dysfunction markers as the calcification of atherosclerotic foci increased.

Next, we performed multivariate logistic regression analysis with the construction of models where the parameter “stable/unstable atherosclerotic plaque” was a dependent variable. We found that the probability of unstable plaque presence inversely correlates with the plaque content of E-selectin (Exp(B) = 0.924, 95% confidence interval (CI) 0.854–0.999, *p* = 0.047) and directly correlates with the calcification degree of the atherosclerotic focus. For example, in the presence of small calcification deposits, the probability of development of an unstable plaque was 4.4-fold higher (Exp(B) = 4.413, 95% CI 1.545–12.602, *p* = 0.006) and 39.4-fold higher in the presence of large calcification deposits in it (Exp(B) = 39.443, 95% CI 3.564–436.53, *p* = 0.003). We also conducted multivariate logistic regression analysis with the construction of models where the parameter “calcification in the plaque is present/absent” was the dependent variable. We noticed that relative risk of calcification formation in atherosclerotic plaques of coronary arteries was associated with osteocalcin (Exp(B) = 1.011, 95% CI 1.004–1.018, *p* = 0.001).

To understand the pathogenesis features of an atherosclerotic lesion in the vascular wall that lead to the formation of an unstable plaque, it is necessary to consider not only local but also systemic processes. Therefore, assays performed in parallel on the vessels and on the blood are relevant and important in this context. In our assessment of correlations of the studied parameters between atherosclerotic plaques and blood, significant correlations were found for sVCAM-1 (*r* = 0.180, *p* = 0.038), MCP-1 (*r* = 0.263, *p* = 0.003), and osteocalcin (*r* = 0.353, *p* = 0.0001). These results point to the feasibility of these markers’ assays in the blood for evaluating the state of atherosclerotic plaques.

Furthermore, to detect biomarkers in the blood that are significantly characteristic of unstable plaques, all blood samples were distributed into two groups: (1) blood samples that corresponded to tissue samples containing stable plaques (according to histological analysis), and (2) blood samples that corresponded to tissue samples containing unstable plaques (Table 4). No significant differences were detectable between these groups.

Likewise, to identify biomarkers in the blood that are significantly characteristic of atherosclerotic plaque calcification, all blood samples were distributed into two groups: (1) blood samples that corresponded to tissue samples containing calcified plaques (according to histological analysis), and (2) blood samples that corresponded to tissue samples containing plaques without calcification (Table 5).

**Figure 4:**
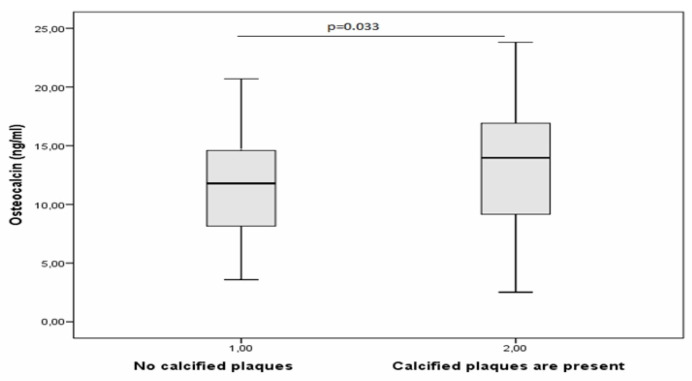
Concentration of osteocalcin in the blood of males with coronary atherosclerosis, depending on the presence of calcification deposits in atherosclerotic plaques.

Among all the studied parameters, only osteocalcin showed a significant difference between the two groups (Figure 4). In males with calcified plaques in coronary arteries, blood osteocalcin concentration was 1.2-fold higher.

These results also indicate the suitability of osteocalcin assays in the blood for evaluating the state of atherosclerotic plaque calcification.

## 4. Discussion

In this study, unstable plaques (which are the cause of adverse cardiovascular events) turned out to be more calcified. In a multivariate logistic regression analysis, we found that the risk of development of an unstable plaque is associated with the degree of calcification of the lesion. Our results are consistent with the finding of Hoffmann et al. that coronary-artery calcium is most strongly associated with CHD, regardless of other cardiovascular risk factors, and that an increase in the arterial calcium level increases the risk of adverse events [5].

When examining markers of endothelial dysfunction, we noted that unstable plaques and plaques with large calcification deposits contain lower levels of sVCAM-1 and E-selectin. Our data are in agreement with several studies. For instance, Oishi et al. [6] revealed that the level of E-selectin was highest in the group of patients with moderate atherosclerosis and stable CHD, in contrast to the group of patients with well-pronounced atherosclerosis. In their study, Jang et al. [7] emphasized that E-selectin is especially important at early stages of atherosclerosis development. Increased blood concentrations of E-selectin—which is expressed by cells of a damaged endothelium and helps to recruit leukocytes—lead to greater endothelial damage, atherosclerosis progression, and the development of cardiovascular diseases. According to a study by Kunutsor et al., the blood level of sVCAM-1 is inversely and independently associated with cardiovascular diseases [8]. Hulok et al. demonstrated that an increased blood level of sVCAM-1 is a predictor of acute coronary syndrome [9]. It was also reported that an increased blood level of sVCAM-1 is an indicator of the presence of atherosclerosis in coronary arteries but not its progression [10]. Thus, the results of our and other studies indicate that E-selectin and sVCAM-1 are significant factors of early stages of coronary atherosclerosis and of atherosclerotic plaque calcification.

In our analysis of calcification markers, unstable plaques manifested a lower osteonectin level and insignificant downregulation of osteoprotegerin and osteopontin in comparison with stable plaques. In addition, plaques with large calcification deposits featured lower concentrations of osteonectin and osteopontin and insignificant downregulation of osteoprotegerin. In the discussion of these results, it is important to point out that the findings about these three calcification-related biomolecules are contradictory among different studies. For example, judging by the results of Lin at al., high blood levels of osteoprotegerin and osteopontin are strong predictors of mortality in patients with CHD [11]. Tousoulis et al. showed that serum levels of osteoprotegerin and osteopontin positively correlate with arterial stiffness and CHD [12]. Those authors suggested that the levels of osteoprotegerin and osteopontin are significantly related to vascular function, thereby contributing to the pathogenesis of atherosclerosis [12]. On the other hand, according to Callegari et al., osteoprotegerin inhibits vascular calcification [13], in line with our data. In a study, Gadeau et al. found no osteonectin in calcification deposits in blood vessels, while osteopontin and osteocalcin were detectable in more mature calcified plaques [14]. Those authors proposed that osteopontin, osteocalcin, and osteonectin are not involved in the initiation stage of the calcification process, but osteopontin and osteocalcin may play a role in the regulation of arterial calcification [14]. On the contrary, Ciceri et al. hypothesized that osteonectin can play a regulatory role in the calcification process and exerts a potentiating action in the regulation of mitosis and cell differentiation [15]. Hirota et al. suggested that osteopontin participates in the calcification of atheromatous plaques because atheromatous plaques are surrounded by macrophages expressing osteopontin messenger RNA (mRNA), and its level increases with the progression of the atherosclerotic focus [16].

Lastly, the results of our study indicate a major role of osteocalcin as a biomolecule that potentiates the development of a calcified unstable plaque. International literature data about the effects of osteocalcin on vascular calcification and stabilization/destabilization of atherosclerotic plaques are contradictory. For instance, the results of Millar et al. imply that osteocalcin is not a mediator of vascular calcification [17]. Rashdan et al. showed the colocalization of osteocalcin with the calcification of smooth muscle cells in calcified plaques and pointed out the crucial role of osteocalcin in arterial calcification [18]. In their review, Tacey et al. theorized that osteocalcin has a protective effect on endothelial function by preventing the development of atherosclerosis, but whether osteocalcin affects vascular calcification remains unclear [19]. Zhang et al. [20] demonstrated a positive correlation between the number of endothelial progenitor cells carrying osteocalcin and coronary-vessel calcification in patients with CHD. In a study, Foresta et al. concluded that platelets in the area of an atherosclerotic plaque additionally release osteocalcin into the plaque [21].

## 5. Conclusions

In general, our results suggest that the calcification of an atherosclerotic plaque promotes its transition to an unstable state and that osteocalcin performs an important potentiating function in these processes. Here, we obtained the same results when assaying the concentration of this biomolecule in the blood; specifically, the concentration of osteocalcin in the blood directly correlates with its content in atherosclerotic plaques and is significantly higher in the blood of male patients having calcified plaques in the arteries. Our findings provide the basis for further research on the suitability of osteocalcin as a potential biomarker of an unstable calcified atherosclerotic plaque in a coronary artery.

## Figures and Tables

**Table 1:** Clinical characteristics of patients with coronary atherosclerosis.

Parameter	Value
Age, yeas (M ± SD)	60.6 ± 7.8
Systolic blood pressure (M ± SD)	137.9 ± 15.5
Diastolic blood pressure (M ± SD)	84.2 ± 9.5
Pulse, rate (M ± SD)	68.9 ± 7.11
Body mass index, kg/m^2^ (M ± SD)	29.2 ± 4.9
History of myocardial infarction	69.4%
History of diabetes type 2	11.9%
Multivascular atherosclerotic lesion of coronary arteries (more than two vessels)	90.1%
History of angina pectoris:	
Funcional class I	0%
Funcional class II	10.9%
Funcional class III	82.6%
Funcional class IV	6.5%

**Table 2:** A comparison of calcification markers and endothelial-dysfunction markers between stable and unstable atherosclerotic plaques (median (interquartile range): Me (25%; 75%)).

Indicator	Stable Plaques (*n* = 89)	Unstable Plaques (*n* = 51)	*p*
Osteoprotegerin, pg/mg protein	110.7 (43.0; 235.9)	89.9 (34.4; 249.4)	0.491
Osteopontin, ng/mg protein	3.3 (1.4; 8.1)	1.7 (0.7; 7.6)	0.120
Osteocalcin, ng/mg protein	16.9 (2.3; 112.9)	14.0 (3.7; 100.5)	0.691
Osteonectin, μg/mg protein	2.4 (1.4; 4.4)	1.6 (1.1; 2.9)	**0.024**
MCP-1, pg/mg protein	79.0 (35.4; 169.5)	85.4 (35.5; 174.1)	0.904
sVCAM-1, ng/mg protein	122.9 (45.9; 254.4)	66.4 (18.5; 161.3)	**0.014**
E-selectin, ng/mg protein	5.43 (1.8; 14.9)	2.02 (1.1; 3.8)	**0.001**

Number in bold are statistically significant in comparisons.

**Table 3:** Markers of calcification and of endothelial dysfunction in plaques showing different degrees of calcification (Me (25%; 75%)).

Indicator	(1) No Calcification Deposits (*n* = 59)	(2) Small and Dust-like Calcification Deposits (*n* = 67)	(3) Large Calcification Deposits (*n* = 14)	Statistical Significance
Osteoprotegerin, pg/mg protein	122.4 (43.8; 194.7)	115.1 (32.3; 264.0)	66.7 (40.4; 118.5)	*p* > 0.05
Osteopontin, ng/mg protein	4.6 (1.8; 10.2)	2.3 (0.9; 7.3)	1.1 (0.3; 3.5)	1 vs. 2 (*p* = 0.05) 1 vs. 3 (*p* = 0.012)
Osteonectin, μg/mg protein	2.3 (1.1; 4.9)	2.0 (1.3; 3.8)	1.0 (0.4; 2.1)	1 vs. 3 (*p* = 0.016) 2 vs. 3 (*p* = 0.023)
MCP-1, pg/mg protein	80.2 (35.0; 147.8)	77.5 (37.2; 174.1)	148.2 (29.0; 179.3)	*p* > 0.05
sVCAM-1, ng/mg protein	115.0 (44.5; 239.2)	110.9 (34.3; 229.5)	30.3 (8.1; 52.3)	1 vs. 3 (*p* = 0.005) 2 vs. 3 (*p* = 0.01)
E-selectin, ng/mg protein	4.8 (2.1; 13.0)	3.1 (1.1; 7.9)	0.9 (0.2; 2.1)	1 vs. 2 (*p* = 0.016) 1 vs. 3 (*p* = 0.002) 2 vs. 3 (*p* = 0.048)

**Table 4:** Biochemical parameters of calcification and of endothelial dysfunction in the blood of males with coronary atherosclerosis, depending on plaque stability (Me (25%; 75%)).

Parameter	Stable Plaques in Coronary Arteries	An Unstable Plaque Is Present in Coronary Arteries
Osteoprotegerin (pg/mL)	60.5 (36.5; 79.9)	49.0 (43.5; 60.5)
Osteopontin (ng/mL)	28.2 (18.12; 42.1)	27.5 (17.1; 38.0)
Osteocalcin (ng/mL)	12.0 (8.3; 16.5)	14.6 (7.8; 17.9)
Osteonectin (µg/mL)	8.9 (8.0; 10.9)	9.2 (7.5; 10.4)
MCP-1 (pg/mL)	404.6 (283.9; 530.9)	547.4 (353.9; 625.4)
sVCAM-1 (ng/mL)	838.8 (669.5; 1023.1)	809.2 (655.6; 935.8)
E-selectin (ng/mL)	47.7 (33.3; 60.2)	54.5 (38.2; 62.1)

**Table 5:** Biochemical indicators of calcification and of endothelial dysfunction in the blood of males with coronary atherosclerosis, depending on the presence of calcification deposits in atherosclerotic plaques (Me (25%; 75%)).

Indicator	No Calcified Plaques	Calcified Plaques Are Present
Osteoprotegerin (pg/mL)	59.4 (33.9; 78.9)	52.0 (41.7; 78.4)
Osteopontin (ng/mL)	8.7 (7.2; 10.8)	9.2 (8.0; 10.422)
Osteonectin (µg/mL)	8.7 (7.2; 10.8)	9.2 (7.9; 10.4)
MCP-1 (pg/mL)	421.1 (294.7; 563.8)	467.7 (398.9; 622.8)
sVCAM-1 (ng/mL)	843.5 (695.7; 1003.6)	838.8 (669.5;1023.1)
E-selectin (ng/mL)	41.8 (33.4; 60.2)	53.7 (38.9; 62.1)
